# *Aeromonas hydrophila* Sepsis Associated with Consumption of Raw Oysters

**DOI:** 10.1155/2014/163040

**Published:** 2014-11-19

**Authors:** Ivan Nikiforov, John Goldman, Pramil Cheriyath, Anix Vyas, Vinod Nookala

**Affiliations:** Department of Medicine, PinnacleHealth System, Harrisburg Hospital, BMAB 3-C, 207 South Front Street, Harrisburg, PA 17104, USA

## Abstract

*Introduction. Aeromonas hydrophila* is a gram negative bacillus that is native to aquatic environments that is increasingly reported in humans. This case is remarkable for *A. hydrophila* with an initial presentation of acute pancreatitis. *Case Presentation.* A 61-year-old male presented to the emergency department with nausea, vomiting, and abdominal pain for two days. His past medical history was significant for alcohol abuse. Initial laboratory examination showed an elevated white blood cell count, elevated lipase, and elevated liver function tests (LFT). Computer tomography (CT) showed peripancreatic inflammatory changes and retroperitoneal free fluid, suggestive of acute pancreatitis. The patient was treated with intravenous (IV) fluids and IV meropenem. After two days, the patient developed sepsis and respiratory failure and was intubated. Blood cultures were positive for *Aeromonas hydrophila* sensitive to ciprofloxacin which was added to his treatment. Additionally, it was discovered that this patient had recently vacationed in Florida where he consumed raw oysters. He was discharged home on the eighth day of the hospital admission. *Conclusion.* This is a rare case of *A. hydrophila* sepsis in an elderly patient with acute pancreatitis and a history of consumption of raw oysters. This case suggests that *A. hydrophila* can cause disseminated infection in immunocompetent individuals.

## 1. Background

The infection of* Aeromonas* is often associated with consumption of fish, shellfish, and open wound exposure to contaminated water. In humans, it can cause a wide variety of infections including gastroenteritis, necrotizing fasciitis, myonecrosis, cellulitis, pneumonia, empyema, and urogenital tract infections [1].* Aeromonas* has also been associated with septicemia in immunocompromised patients or those who have experienced acute injury in an aquatic environment allowing the bacteria to be introduced from an outside source. A small, but growing, subset of* Aeromonas* septicemia cases have been reported in individuals with no risk factors [1]. In this rare case, we report* Aeromonas hydrophila* septicemia associated with the consumption of raw oysters in a 61-year-old male with the initial presentation of acute pancreatitis.

## 2. Case Presentation

A 61-year-old male presented to the emergency room with the complaint of nausea, vomiting, and abdominal pain that began the previous day. Patient denied fever, diarrhea, constipation, bloody vomiting, or blood in the stool. His past medical history was only significant for alcohol abuse and smoking. The patient was found to be in no acute distress. Vital signs were stable except for tachycardia at 121 beats per minute. Physical examination showed mild abdominal distention, tenderness, hypoactive bowel sounds, and guarding. Laboratory examination revealed hemoglobin of 19.5 g/dL, leukocytes of 20,200 cells/mm^3^, lipase of 789 U/L, AST of 557 IU/L, ALT of 152 IU/L, and total bilirubin of 9.1 mg/dL. Patient underwent CT scan of the abdomen which showed peripancreatic inflammatory changes and retroperitoneal free fluid, suggestive of acute pancreatitis. Due to concern of progression to pancreatic necrosis, the patient was admitted to the ICU, made NPO, and given IV fluids. The next day the patient's condition rapidly deteriorated, he became unresponsive, and he developed respiratory failure requiring intubation. IV meropenem 500 mg every 8 hours was started empirically. On the third day of the hospital admission, the blood cultures returned positive for* A. hydrophila* which were isolated from both aerobic and anaerobic vials. The specimen was cultured on blood agar and chocolate agar. Gram stain showed gram negative bacilli. Additional tests were done via MicroScan system which showed a 91% probability of* A. hydrophila* group. Test for* Vibrio cholera* was negative. Sequencing via* rpoD* gene confirmed* A. hydrophila*.* Aeromonas* was found to be sensitive to ciprofloxacin. IV ciprofloxacin 400 mg once daily was added to his antibiotic treatment. Additional history was obtained from the family, and it was discovered that the patient was vacationing in Florida two weeks previously, where he ingested raw oysters. The patient recovered and was discharged on the eight day of the hospital admission.

## 3. Discussion

*Aeromonas* are gram negative facultative anaerobes, which are indigenous to aquatic environments and are known for causing infection in fish and animals. Their morphological characteristics include straight cocobacillary to bacillary cells with an approximate diameter of 1.0 micrometer, and a length of 3.5 micrometers.* Aeromonas* are usually motile with a single polar flagellum [2]. Recently, there were increased reports of debilitating infections in humans including fatal septicemia. In 2004, it was estimated incidence of* Aeromonas* septicemia was 1.5 per million population [1]. As of 2009, there were 22 recorded species of* Aeromonas* with 2 new species being proposed [3]. The following species are associated with human clinical infection:* Aeromonas hydrophila, A. aquariorum, A. veronii, A. caviae, A. trota*, and* A. media* [3, 4].

Most* Aeromonas* are isolated in the laboratory using blood agar with 20 *μ*g of ampicillin with the exception of* A. trota* due to its sensitivity to ampicillin [5]. Further identification is done based on the beta-hemolysis by screening with the oxidase and spot indole test. In order to distinguish* Aeromonas* form* Vibrio* and* Plesiomonas* the culture is grown in salt concentrations >6% with vibriostatic agent O/129 [2]. Gene sequencing is the best way to differentiate* Aeromonas* on a species level. The sequencing of 16S rRNA, a single housekeeping gene, was previously used to differentiate* Aeromonas* species [6]. Unfortunately, it turned out that horizontal transfer of the gene occurred between species making the results unreliable. The* rpoD* and* gyrB* genes have been far more precise in identifying* Aeromonas* species [4]. If long term storage of the isolate is needed, the recommendation is to use trypticase soy broth with a 30% glycerol at a temperature of −80 centigrade [7].

The pathogenicity of* A. hydrophila* is caused by a type III secretion system (TTSS) and* act* gene [8, 9]. TTSS enables the delivery of toxins inside the host cells. A type II-secreted cytotoxic enterotoxin (Act) which has hemolytic and cytotoxic functions is encoded by the* act* gene. These virulence factors are linked to clinical infection and play a key role in the disease process.* A. hydrophila* also has the enzymes elastase and pectinaseare. These enzymes act on the immune system and cause tissue destruction and hemolysis [10]. The majority of* Aeromonas* infections usually present as gastroenteritis with the symptoms of acute watery diarrhea, abdominal pain, fever, vomiting, and nausea. The infections are self-limiting with mild dehydration.* Aeromonas* infections are also known to cause hemolytic urmeic syndrome, meningitis, peritonitis, wound infections, respiratory tract infection, ocular diseases, cellulitis, necrotizing fasciitis, peritonitis and cholangitis. Near drowning events have led to* Aeromonas* pneumonia [1]. Our extensive literature review did not find a single report of sepsis with the initial presentation of acute pancreatitis. Overall, there are no clinical symptoms that clearly help to distinguish* Aeromonas* sepsis from sepsis caused by other bacteria, which is unfortunate since* Aeromonas* septicemia can have a mortality rate as high as 46% [11].

The main treatment of* Aeromonas* is quinolones with levofloxacin and ciprofloxacin being the antibiotics of choice. There are reports of resistance to quinolones due to the qnr protein. This product interferes with the DNA gyrase inhibitor of quinolones and grants resistance to them [12]. If the qnr protein is present, additional antibiotic coverage with trimethoprim-sulfamethoxazole or cephalosporins should be considered. Due to the above concerns, current recommendation does not include starting the patient on empiric antibiotic treatment. Clinicians should wait for culture and sensitivity results before antibiotic treatment is started.* Aeromonas* possess a wide range of beta-lactamases, generally making them resistant to penicillins, cephalosporins, extended spectrum cephalosporins, clavulanic acid, tazobactam, and sulbactam [13].

## 4. Conclusion

Although* Aeromonas* septicemia is rare, it can carry a high mortality rate of 46%. Immunocompromised patients, those who suffered open wounds in aquatic environments and those who have consumed fish and shellfish are at highest risk. There has been an increasing amount of* Aeromonas* infections in immunocompetent patients. Since there is wide spread consumption of raw fish and shellfish in the United States, clinicians should consider* Aeromonas* infection in addition to* Vibrio* infection as one of the differential diagnoses.
